# Postnatal ethanol exposure impairs social behavior and operant extinction in the adult female mouse offspring

**DOI:** 10.3389/fnins.2023.1160185

**Published:** 2023-05-16

**Authors:** Sebastiano Bariselli, Noa Reuveni, Nina Westcott, Yolanda Mateo, David M. Lovinger

**Affiliations:** Laboratory for Integrative Neuroscience (LIN), National Institute on Alcohol Abuse and Alcoholism, Bethesda, MD, United States

**Keywords:** late-term gestational ethanol exposure, social behavior, anhedonia, extinction, anxiety-like behaviors

## Abstract

Fetal Alcohol Spectrum Disorder (FASD) comprises a group of neurodevelopmental deficits caused by alcohol exposure during pregnancy. Clinical studies suggest that while the male progeny experiences serious neurodevelopmental defects, female patients have more severe cognitive, social, and affective symptoms. Other than sex, dose, frequency, and timing of exposure determine the neurobehavioral outcomes in young and adult progeny. In this regard, human studies indicate that some individuals relapse during late-term gestational periods. In mice, this interval corresponds to the first 10 days after birth (postnatal, P0-P10). In our model of postnatal ethanol exposure (PEE^P0-P10^), we tested whether adult female and male offspring show deficits in sociability, anxiety-like, reward consumption, and action-outcome associations. We report that female PEE^P0-P10^ offspring have mild social impairments and altered extinction of operant responding in the absence of anxiety-like traits and reward consumption defects. None of these deficits were detected in the male PEE^P0-P10^ offspring. Our data provide novel information on sex-specific neurobehavioral outcomes of postnatal ethanol exposure in female adult offspring.

## Introduction

Fetal Alcohol Spectrum Disorder (FASD) is a class of heterogeneous neurobehavioral deficits caused by alcohol exposure during pregnancy, with current estimates indicating a prevalence of 71.4 out of 1,000 children (May et al., 2021; Glass et al., 2023) and high socio-economic costs (Ericson et al., 2017; Greenmyer et al., 2018). FASD clinical symptoms are heterogenous and include anatomical, motor, cognitive, and socioemotional abnormalities (Mattson et al., 2011, 2019; Riley et al., 2011; Hoyme et al., 2016). Although many clinical studies indicate that prenatal exposure to drugs, including alcohol, primarily affects male individuals (Traccis et al., 2020), the prevalence of FASD among sexes shows great variability across different patient cohorts. While some authors report a higher incidence of FASD among male individuals (May et al., 2000; Burd et al., 2003), others find a similar prevalence between sexes (Palmeter et al., 2021) or an even higher incidence of FASD diagnosis in the female subpopulation (Autti-Rämö et al., 2006). Moreover, male subjects have reduced survivability and heightened neurodevelopmental impairments when exposed to prenatal binge-like alcohol levels compared to females (May et al., 2017; Flannigan et al., 2023). On the other hand, female FASD individuals display more severe cognitive, social, and affective symptoms (May et al., 2017; Flannigan et al., 2023). However, considering that most preclinical studies focused on the effects of fetal alcohol exposure on male progeny (Terasaki et al., 2016), whether female subjects show cognitive and socioemotional defects remains a fundamental research question. The answer will improve our understanding of whether biological sex interacts with alcohol to determine FASD severity and might ultimately help develop and tailor therapeutic strategies to specific subgroups of FASD individuals.

Besides the sex of the progeny, the dose and the frequency of maternal alcohol use influence the neurobehavioral outcomes of fetal alcohol exposure (Maier and West, 2001; May et al., 2014). Frequent maternal ingestion of large quantities of alcohol has been associated with higher severity of FASD symptoms (Maier and West, 2001; May and Gossage, 2011). For example, gestational binge-like levels of alcohol have been linked to more pronounced anatomical dysmorphology (Patra et al., 2011). During pregnancy, the fetus goes through various stages of organ formation and development; thus, the timing of alcohol exposure also largely influences the outcomes of FASD (May and Gossage, 2011). Considering that the human brain develops during pregnancy, the heterogeneity of FASD neurobehavioral symptoms might depend, at least partly, on the timing of alcohol exposure and the resulting developmental deficits in discrete brain regions (Guerri et al., 2009; Bariselli and Lovinger, 2021). Animal models of fetal alcohol exposure consider that the first ten embryonic days (E0-E10) corresponds to the first trimester of pregnancy, the second half of rodent pregnancy (E11-E20) to the second trimester, and the first 10–14 postnatal days (P0-P10/P14) to late-term gestational periods (Marquardt and Brigman, 2016). This reflects a different timing of neurodevelopmental milestones between rodents and humans. Events such as blood–brain barrier formation and increased axonal and dendritic density primarily occur within the uterus in humans and during early postnatal periods in rodents (Semple et al., 2013). Clinical evidence indicates that most women stop alcohol use once the pregnancy is ascertained, while 27% report alcohol use episodes throughout pregnancy (Muggli et al., 2016). In other studies, 40% of pregnant women reported alcohol drinking during the last trimester of gestation (Little et al., 1990), while about 30–50% of those who remained abstinent during pregnancy relapsed during the three-month post-parturition period (Jagodzinski and Fleming, 2007; Forray et al., 2015). These clinical data highlight the importance of investigating the behavioral outcomes of alcohol exposure during late-term gestational periods in adult progeny.

Individuals diagnosed with FASD (Kully-Martens et al., 2012) and animal models of fetal alcohol exposure display social deficits across their lifespan; prenatal and post-natal alcohol exposure impairs mother-pup interactions (Barron et al., 1991; Subramanian, 1992) and play behavior during adolescence in both sexes (Meyer and Riley, 1986). During adulthood, models of prenatal alcohol exposure display altered sexual maturation in male and female offspring (Creighton-Taylor and Rudeen, 1991; Mcgivern et al., 1992), with sexually dimorphic alterations in direct social interaction in the adult progeny (Kelly et al., 1994). Postnatal ethanol exposure (PEE) produces social interaction deficits in adolescent and adult male rats (Boschen et al., 2014) and social recognition deficits in adult male mice during dyadic conspecific interaction tests (Subbanna et al., 2018; Joshi et al., 2019; Shivakumar et al., 2021). In addition to dyadic social interaction, the social preference assay is widely used to characterize sociability and social preference in mouse models of neurodevelopmental disorders (Yang et al., 2011; Rein et al., 2020). However, whether male and female PEE mice display social preference and social approach deficits remains an open question.

In addition to social deficits, FASD is often associated with the appearance of psychiatric conditions, including anxiety and depression (Famy et al., 1998; Barr, 2006; Pei et al., 2011). Animal models of prenatal ethanol exposure show anxiety-like behavior in male and female progeny during elevated-plus or O-maze testing (Dursun et al., 2006; Cullen et al., 2013; Oubraim et al., 2022), with some experiments reporting anxiety-like traits in males only (Rouzer et al., 2017). PEE does not affect elevated plus-maze exploration (Gibula-Tarlowska et al., 2021) but impairs passive avoidance in adult male rats (Lopatynska-Mazurek et al., 2021b). Additionally, female PEE rats displayed a reduced time spent in the center of an open field compared to controls (Bianco et al., 2021). Prenatal ethanol exposure offspring also leads to heightened learned helplessness in the shuttle-box test and increased immobility time in the forced-swim task (Caldwell et al., 2008), originally developed to assess anti-depressive actions of pharmacological interventions (Porsolt et al., 1978). These deficits are associated with heightened sucrose responsivity in male mice (Hellemans et al., 2010) measured in the two-bottle choice test. Other studies found no difference (Sanchez Vega et al., 2013; Yu et al., 2020). Whether PEE induces anxiety-like and depressive-like states in the female and male adult mouse offspring remains under-investigated.

Laboratory animals used as models of FASD display deficits in associative learning and executive function (reviewed in Bariselli and Lovinger, 2021) throughout their lifespan. Prenatal exposure and PEE affect the acquisition and reversal of contextual associations during the T-, Y-Maze, Barnes Maze and Morris Water Maze tasks in male and female adolescent rats (O’Leary-Moore et al., 2006; Allan et al., 2014; Gibula-Tarlowska et al., 2021; Lopatynska-Mazurek et al., 2021a; Risbud et al., 2022), and impair contextual pre-exposure facilitation of fear learning (Heroux et al., 2019). During adulthood, PEE alters spatial associative learning in male and female mice (Subbanna et al., 2018; Joshi et al., 2019; Shivakumar et al., 2021). Instrumental learning tasks also revealed that prenatal exposure and PEE increase lever pressing under specific training schedules and interferes with habitual responding in the adult offspring (Cuzon Carlson et al., 2020). Changes in action strategy lead to behavioral maladaptations upon changes in cue-reward contingencies in both prenatally exposed and PEE male and female offspring (Marquardt et al., 2014; Gursky et al., 2021) and alter cue-mediated reinstatement in the progeny of both sexes (Olguin et al., 2019). However, whether PEE impairs the extinction of action-outcome associations in the adult offspring remains an open question.

Considering the impact of timing and sex in determining the neurobehavioral outcomes of fetal alcohol exposure, we aimed to characterize the neurobehavioral outcomes of binge-like PEE on the socio-emotional and cognitive behavioral domains during adulthood. In adult female offspring, we demonstrate that alcohol exposure during the equivalent of the third trimester induces mild impairments in social and cognitive function without causing major anxiety-like or reward-processing defects. These data help characterize the influence of the timing of developmental ethanol exposure on specific behavioral symptoms in a sex-specific subgroup of PEE subjects.

## Methods

### Experimental subjects

Pregnant C57Bl6/J WT female mice were purchased at embryonic day 7 (E7) from the Jackson Laboratory. The animals were acclimated to the procedure room for 3–4 days before pup delivery. Their progeny underwent air (CE^P0-P10^) or postnatal ethanol exposure (PEE^P0-P10^) between P0-P10 and were weaned at P21. The behavioral experiments described in this work were conducted on the adult male and female progeny of five PEE cohorts and their CE controls; cohorts #1 and #2 were used for operant training, while cohorts #3, #4, #5 were used for social preference, O-Maze, and sucrose preference assays. Animals were maintained on a 12-h dark/12-h light cycle for the whole duration of the experiments. Mice were treated in accordance with the *NIH Guide for the Care and Use of Laboratory Animals*. The data were collected through experimental procedures approved in the LIN-DL-1 protocol for animal authorization by the Animal Care and Use Committee of the NIAAA Division of Intramural Clinical and Biological Research.

### Postnatal ethanol exposure protocol

At P0, the home cages with dams and pups were placed in air-tight plexiglass chambers. 190-proof EtOH was vaporized at a rate of 8–9 liter of air/min and adjusted to reach a concentration between 0.1–0.15 mg/dL of EtOH in the air. Pups and dams were exposed to EtOH (PEE) or air (CE) in a 16-h-ON/ 8-h-OFF cycle for 10 days, with a 3-day break. Ethanol and air exposures started between 5–6 p.m. and terminated at 9–10 a.m. Blood Alcohol Concentration (BAC) was measured from trunk blood collected after pup decapitation. Serum was obtained, diluted, and alcohol concentration was measured using a colorimetric assay (Pointe Alcohol Reagent Test).

### Pup retrieval assay

Upon removing dams and pups from the vapor chambers, we conducted a pup retrieval assay to evaluate maternal behavior. CE^P0-P10^ and PEE^P0-P10^ dams were placed in a home cage-like arena with their litter and nest for at least 5 min for habituation. Afterward, we performed a pup retrieval assay by removing one pup at a time from their nest for 10 consecutive trials with no breaks in between trials. One trial began upon placing a pup on the opposite corner of the arena relative to the nest. The latency to pup retrieval was measured as the time between pup removal from the nest by the experimenter and nest placement by the dam. Each trial lasted a maximum of 120 s. This time limit was based on previous studies using pup retrieval assay (Marlin et al., 2015; Carcea et al., 2019).

### Three-chamber sociability task

During adulthood (P70-P260), male and female mice from cohorts #3, #4, and #5 underwent a three-chamber assay in a black-walled plexiglass arena divided into three chambers. The social and object chambers (18 cm x 20 cm) contained an enclosure with or without a sex-matched (either male or female) younger conspecific (6–8 weeks of age). The two chambers were connected through a smaller corridor (20 cm x 10 cm). During the habituation phase, animals were placed in the arena for 10 min. During the sociability phase, animals were briefly confined in the corridor, and the object and the social stimuli (male or female) were inserted in the opposite sides of the arena. As reported in previous studies (Bariselli et al., 2016), during this phase animals were allowed to explore the arena for 10 min. Video recordings were obtained and analyzed using EthoVision software. The animal’s exploratory behavior was automatically scored to avoid camera artifacts and experimenter biases. We delimited a 2–3 cm region around the enclosure (proximal zone), while the rest was considered the distal zone relative to the social stimulus. We automatically scored distance moved, time spent in either chamber and time spent in the proximal or distal zone of the social stimulus. We expressed these data as a percentage of total exploration time.

### Elevated O-maze

One day after completing the three-chamber task, male and female CE^P0-P10^ and PEE^P0-P10^ mice (P70-P260) from cohorts #3, #4, and #5 underwent testing in the elevated O-maze. The circular maze (60 cm diameter) was elevated 50 cm from the ground, with portions enclosed by 16 cm high removable walls on either side. The O-maze area inside the walls was considered “closed,” and the area with only the base and no surrounding walls was considered “open.” After at least 30 min of habituation to the room, one mouse was placed inside a closed area to start and allowed to explore the arena, while recordings were performed with a camera placed above the maze using Bonzai software. Time spent in open areas and distance moved was automatically scored using Ethovision software for video recording analysis for a total of 5 min, as reported in previous studies (Braun et al., 2011). The first 30 s of each video were excluded from the analysis to avoid camera artifacts.

### Sucrose preference test

In the 2 days following the completion of the O-maze, male and female CE^P0-P10,^ and PEE^P0-P10^ (P70-P260) mice from cohorts #3, #4, and #5 underwent a sucrose preference test in their home cage. Mice were separated and placed in cages with lids equipped to hold two sipper bottles. Both sipper tubes were filled with regular tap water for the habituation phase, and mice were allowed uninhibited drinking for 5 h. At the end of the day, sipper bottles were exchanged for one water bottle identical to the habituation bottle and one sipper bottle filled with 1% sucrose. All bottles were weighed beforehand, and cages were counterbalanced for which sipper bottle contained sucrose (left or right). Sixteen hours later, on the morning of the following day, bottles were removed and weighed. Mice received two new bottles with only tap water for 8 h. At the end of the day, the same protocol was carried out except with 8% sucrose, and the location of the sucrose bottles was switched from the previous day. The next morning, bottles were weighed, and mice were re-housed in their original groups. Our 16-h 1 and 8% sucrose preference test is based on previous reports of a duration of the test between 1 and 24-h (Bariselli et al., 2016; Hoffman, 2016). Liquid consumption was normalized to each animal’s body weight (g_consumed_/kg_bodyweight_). Preference for sucrose over the water was determined by dividing water consumed (g/kg) by sucrose consumed (g/kg).

### Operant training

Male and female CE^P0-P10^ and PEE^P0-P10^ experimental subjects (P55-P80) from cohorts #1 and #2 were food restricted to 85–95% of their baseline body weight 3–5 days before the beginning of operant training and throughout the entire behavioral protocol. Subjects were handled for 3–5 min for 3–5 days before the start of the experiments. On day 0 (shaping), subjects were placed in the operant box (MedAssociates), and reward delivery (20% sucrose solution) occurred at random intervals every 60 s on average.

#### Acquisition

During the acquisition phase, from day 1 to day 4, subjects were trained to press a single lever (left or right) to obtain one reward (FR1) consisting of a drop of 20% sucrose delivered in a reward cup. Levers were counterbalanced across experimental subjects. A session ended upon delivery of 30 rewards or when 60 min elapsed (Cuzon Carlson et al., 2020). On day 5, an inactive lever (left or right, counterbalanced) was introduced. Subjects had to press the active lever at FR1 to obtain a maximum of 30 rewards or until 60 min elapsed for 3 consecutive sessions. On day 8, the reward schedule switched from FR1 to FR5 (in which five lever presses were required per reward), and operant conditioning continued for two additional days.

#### Reversal Learning

The day following the last FR5 session, the order of active and inactive levers was switched while maintaining the FR5 schedule of reinforcement. Reversal learning continued for five additional days.

#### Extinction and reinstatement of operant responding

After the last reversal learning session, subjects were tested on a Random Ratio (RR) schedule of reinforcement (Cuzon Carlson et al., 2020). Experimental subjects made an average of 10 (for 2 days), then 20 (for 2 days) active lever presses to obtain a reward, while the other lever remained inactive. The last RR20 session was followed by three extinction sessions, during which RR20 responding was never followed by reward delivery. After the last extinction session, reinstatement of responding was assessed by reintroducing sucrose delivery upon RR20 responding.

Subjects returned to their home cage upon completing each behavioral session. They were fed a grain-based rodent diet (BioServ, F0171) according to their food restriction regime.

### Statistical analysis

For the data reported in this work, each experimental group had a sample size similar to studies previously performed in the laboratory (Cuzon Carlson et al., 2020). Experimental subjects that spent less than 25% of their time in the social chamber (1 CE^P0-P10^ female excluded) or did not acquire lever pressing behavior during operant training (2 PEE^P0-P10^ females excluded, 1 CE^P0-P10^ and 1 PEE^P0-P10^ male excluded) were excluded from the analysis. Subsequently, statistical outliers were identified with the ROUT method (Q = 10%) on object, corridor, and social chamber exploration (none removed), on open arm exploration (1 PEE^P0-P10^ male excluded), consumption of 1% sucrose (1 PEE^P0-P10^ female and 1 PEE^P0-P10^ male excluded), consumption of 8% sucrose (1 PEE^P0-P10^ female excluded), and active lever press at extinction day 1 (2 CE^P0-P10^ and 1 PEE^P0-P10^ female excluded) and removed from the analysis. The normality of sample distribution was assessed with the Shapiro–Wilk test. Two-sample distributions were compared with a two-tailed parametric t-test or non-parametric Mann–Whitney test. Analysis of variance was conducted using repeated measures ANOVA (RM ANOVA), RM two-way ANOVA, or two-way ANOVA followed by *post hoc* tests as reported in each figure graph and legend. Nested analysis of latency to social approach and active lever pressing at extinction day 1 were conducted using a nested t-test that considers each pup a biological replicate of a litter (random factor), while treatment (CE vs. PEE) as a fixed factor. A value of *p* < 0.05 was set to determine the statistical significance of two-sample comparisons, main effects, and interactions. Graphs were created, and statistical analysis was conducted with GraphPad/Prism.

## Results

### Postnatal ethanol exposure alters maternal behavior and female offspring growth

**Figure 1:**
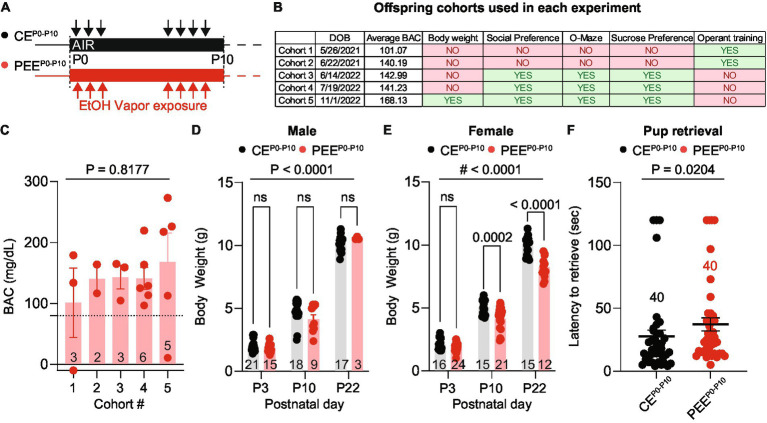
PEE increases BAC and alters offspring growth. **(A)** Ethanol vapor exposure timeline. Pups and dams were exposed to ethanol or air for 16-h sessions during the first 10 postnatal days with a 3-day break. **(B)** Assignment of offspring cohorts used in each experiment and their average blood alcohol concentration (BAC). **(C)** The average BAC in PEE^P0-P10^ pups for each cohort [one-way ANOVA; *F*_(4, 14)_ = 0.3822, *p* = 0.8177]. Number of mice per cohort are indicated on the graph. **(D)** Body weights of CE^P0-P10^ and PEE^P0-P10^ male offspring at postnatal days 3, 10, and 22 [two-way ANOVA; treatment main effect: *F*_(1,77)_ = 0.1801, *p* = 0.6725; time main effect *F*_(2,77)_ = 621.7, *p* < 0.0001; treatment x time interaction *F*_(2,77)_ = 2.633, *p* = 0.0784 followed by Sidak *post hoc* multiple comparison test]. **(E)** Body weights of CE^P0-P10^ and PEE^P0-P10^ female offspring at postnatal days 3, 10, and 22 [two-way ANOVA; treatment main effect: *F*_(1,97)_ = 47.33, *p* < 0.0001; time main effect *F*_(2,97)_ = 896.5, *p* < 0.0001; treatment × time interaction *F*_(2,97)_ = 10, *p* = 0.0001 followed by Sidak *post hoc* multiple comparison test]. **(F)** Latency to pup retrieval in CE^P0-P10^ and PEE^P0-P10^ dams (Mann Whitney test *U* = 560, *p* = 0.0204). Data are expressed as mean ± SEM. Number of mice or trials indicated on each graph.

Our study used a mouse model of postnatal ethanol exposure (PEE^P0-P10^) by exposing dams and pups to ethanol vapor between days P0-P3 and P6-P10 in a 16-h-ON/8-h-OFF pattern (Figure 1A). We included a total of five cohorts of alcohol-exposed dams and pups (Figure 1B): cohorts 1 and 2, with 3 and 2 litters, respectively, were used for the operant training experiments; cohorts 3 and 4, with 3 and 6 litters, respectively, were used for the social preference, two-bottle sucrose preference test, and O-Maze, and cohort 5, with 5 litters, was used to monitor body weight during early development, and all behavioral assays except for operant training. Age-matched air-exposed offspring (CE^P0-P10^) was used for all the behavioral experiments. Between P3 and P9, we sacrificed one pup per litter and measured Blood Alcohol Concentration (BAC) from trunk blood. On average, we detected a BAC above the intoxication threshold of 80 mg/dL, which did not differ across the 5 mouse cohorts (Figure 1C). In cohort 5, we monitored pup growth by measuring their body weights at three developmental time points (P3, P10, and P22). In male PEE^P0-P10^, we did not observe any body weight difference compared to CE^P0-P10^ (Figure 1D). However, PEE^P0-P10^ female pups had a lower body weight at P10 and P22 than CE^P0-P10^ female pups (Figure 1E). We then performed a pup-retrieval assay (Marlin et al., 2015) to investigate whether alcohol vapor exposure impairs maternal behavior. We observed that PEE^P0-P10^ dams had a longer latency to pup retrieval than the CE^P0-P10^ dams (Figure 1F). Altogether, these data indicate that alcohol vapor exposure during postnatal periods affects maternal behavior and female offspring growth in a sex-specific manner.

### Postnatal ethanol exposure induces mild social deficits in the adult female progeny

**Figure 2:**
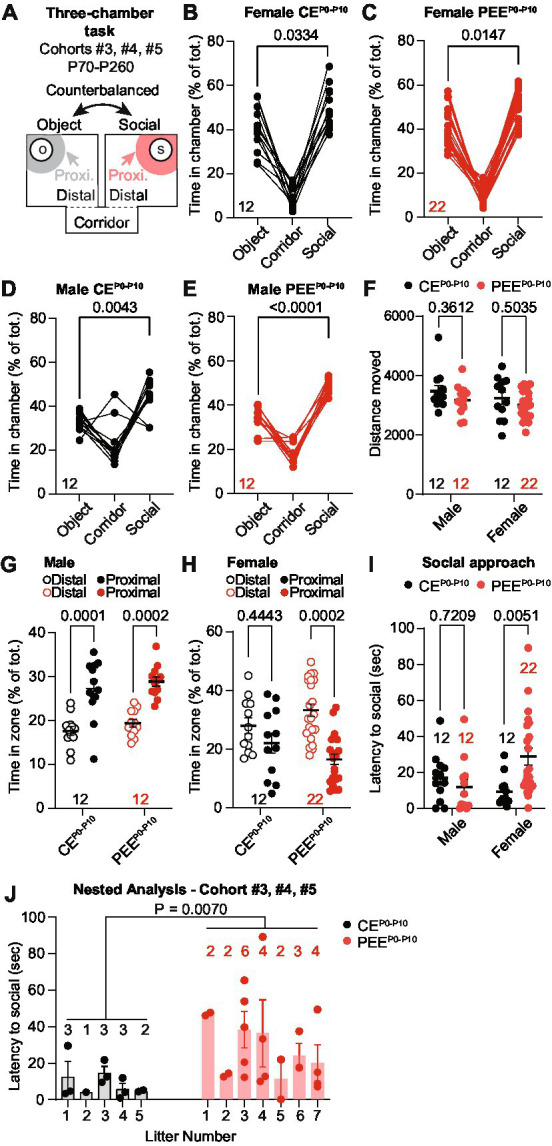
PEE induces mild social deficits in the adult female progeny. **(A)** Schematic diagram of the three-chamber apparatus used in the three-chamber task. **(B)** Percentage of time spent in the corridor, object, and social chamber in CE^P0-P10^ adult female mice (RM ANOVA; *F*_(2,22)_ = 45.93, *p* < 0.0001 followed by Sidak *post hoc* multiple comparison test). **(C)** Percentage of time spent in the corridor, object, and social chamber in PEE^P0-P10^ adult female mice (RM ANOVA; *F*_(1.212, 25.45)_ = 125.1, *p* < 0.0001 followed by Sidak multiple comparison test). **(D)** Percentage of time spent in the corridor, object, and social chamber in CE^P0-P10^ adult male mice (RM ANOVA; *F*_(2,22)_ = 19.04, *p* < 0.0001 followed by Sidak *post hoc* multiple comparison test). **(E)** Percentage of time spent in the corridor, object, and social chamber in PEE^P0-P10^ adult male mice (RM ANOVA; *F*_(1.716, 18.88)_ = 96.11, *p* < 0.0001 followed by Sidak multiple comparison test). **(F)** Distance moved during the sociability assay in male and female CE^P0-P10^ and PEE^P0-P10^ adult progeny (two-way ANOVA; treatment main effect: *F*_(1,54)_ = 2.791, *p* = 0.1006; sex main effect: *F*_(1,54)_ = 1.591, *p* = 0.2127; treatment × sex interaction: *F*_(1,54)_ = 0.0757, *p* = 0.7842). **(G)** Percentage of time spent in distal or proximal areas to social stimulus for CE^P0-P10^ and PEE^P0-P10^ adult male mice (RM two-way ANOVA; zone main effect: *F*_(1,22)_ = 47.83, *p* < 0.0001; treatment main effect: *F*_(1,22)_ = 1.771, *p* = 0.1969, zone × treatment interaction: *F*_(1,22)_ = 0.0035, *p* = 0.9535; followed by Sidak *post hoc* multiple comparison test). **(H)** Percentage of time spent in distal or proximal areas to social stimulus for CE^P0-P10^ and PEE^P0-P10^ adult female mice (RM two-way ANOVA; zone main effect: *F*_(1,32)_ = 12.88, *p* = 0.0011; treatment main effect: F_(1,32)_ = 0.007, *p* = 0.9340, zone × treatment interaction: *F*_(1,32)_ = 2.967, *p* = 0.0946; followed by Sidak *post hoc* multiple comparison test). **(I)** Latency to first social approach in CE^P0-P10^ and PEE^P0-P10^ adult female and male mice (two-way ANOVA; treatment main effect: *F*_(1,54)_ = 2.392, *p* = 0.1278; sex main effect: *F*_(1,54)_ = 1.021, *p* = 0.3168; treatment × sex interaction: *F*_(1,54)_ = 6.946, *p* = 0.0109). **(J)** Nested analysis of the latency to first social approach in CE^P0-P10^ and PEE^P0-P10^ female offspring (nested *t*-test, *t*_(32)_ = 2.883, *p* = 0.007). Data are expressed as mean ± SEM. Number of mice are indicated on each graph.

Previous studies showed that fetal alcohol exposure impairs adolescent and adult progeny sociability. Thus, we assessed whether adult PEE^P0-P10^ offspring display social interaction deficits compared to control exposure (CE^P0-P10^) when given a choice between an unfamiliar same-sex conspecific or an unfamiliar object. These experiments were conducted on the male and female offspring of cohorts #3, #4, and #5 between P70-P260. We used a modified version of the three-chamber sociability assay (Yang et al., 2011). We automatically scored the percentage of time each mouse spent in the corridor, social, and object chambers (Figure 2A). In this task, CE^P0-P10^ and PEE^P0-P10^ female offspring showed a longer time spent in the social compared to the object chamber (Figures 2B,C). Similarly, CE^P0-P10^ (Figure 2D) and PEE^P0-P10^ (Figure 2E) male progeny spent more time in the social, compared to the object, compartment. Distance moved during the social preference assay did not differ between sexes, CE^P0-P10^ and PEE^P0-P10^ offspring (Figure 2F). We then analyzed the exploratory behavior of the animals within the social chamber as time spent in areas proximal or distal to the same-sex unfamiliar social stimulus. We observed that both CE^P0-P10^ and PEE^P0-P10^ male progeny spent a longer time in the proximity of the social stimulus compared to more distal areas (Figure 2G). CE^P0-P10^ female mice did not show differences in exploratory behavior between proximal and distal areas (Figure 2H). In contrast, PEE^P0-P10^ female mice spent more time in the distal than the proximal location relative to the social stimulus (Figure 2H). We then tested whether this different exploratory behavior resulted in changes in social interaction by quantifying the latency to first approach the social stimulus in our experimental groups. While no difference was detected between CE^P0-P10^ and PEE^P0-P10^ male progeny (Figure 2I), we observed that PEE^P0-P10^ female mice had longer delays in interacting with their conspecifics compared to the CE^P0-P10^ group (Figure 2I). To exclude that social approach deficits result from multiple representations of sex and exposure due to the inclusion of more than one pup per litter, we performed a nested analysis that considers each pup a biological replicate of each litter. This per-litter analysis revealed that PEE^P0-P10^ female offspring had a longer latency to approach the social stimulus than CE^P0-P10^ mice (Figure 2J). Thus, while PEE^P0-P10^ adult male and female mice do not have major sociability abnormalities, female PEE^P0-P10^ progeny show a sex-specific deficit in exploratory behavior and longer delays in social approach.

### Postnatal ethanol exposure fails to induce either anxiety-like or reward-processing deficits in the adult female offspring

**Figure 3:**
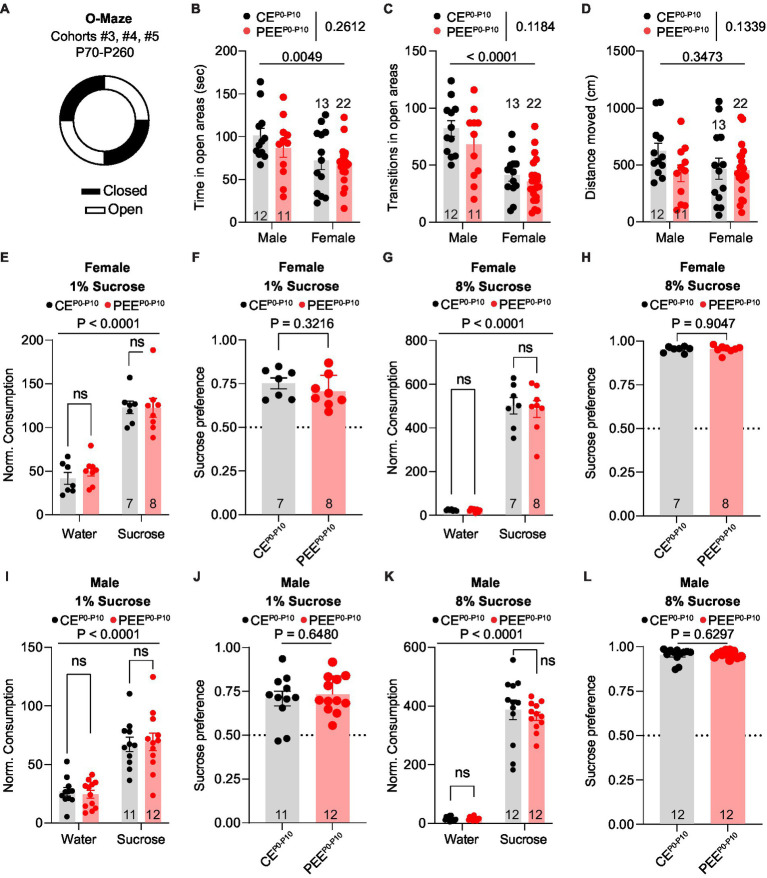
PEE fails to induce anxiety-like and reward consumption deficits in both sexes. **(A)** Schematic diagram of the O-Maze used to assess anxiety-like behavior. **(B)** Time spent in the open areas of the O-Maze in CE^P0-P10^ and PEE^P0-P10^ adult male and female mice (two-way ANOVA; treatment main effect: *F*_(1,54)_ = 1.289, *p* = 0.2612; sex main effect: *F*_(1,54)_ = 8.617, *p* = 0.0049; treatment × sex interaction: *F*_(1,54)_ = 0.3904, *p* = 0.5347). **(C)** Number of open arm transitions in CE^P0-P10^ and PEE^P0-P10^ adult male and female mice (two-way ANOVA; treatment main effect: *F*_(1,54)_ = 2.518, *p* = 0.1184; sex main effect: *F*_(1,54)_ = 35.23, p < 0.0001; treatment × sex interaction: *F*_(1,54)_ = 0.5011, *p* = 0.4821). **(D)** Distance moved for CE^P0-P10^ and PEE^P0-P10^ adult male and female mice (two-way ANOVA; treatment main effect: *F*_(1,54)_ = 2.315, *p* = 0.1339; sex main effect: *F*_(1,54)_ = 0.8988, *p* = 0.3473; treatment × sex interaction: *F*_(1,54)_ = 1.757, *p* = 0.1906). **(E)** Normalized consumption (g/Kg) of sucrose and water consumed during the 1% sucrose preference test in CE^P0-P10^ and PEE^P0-P10^ adult female mice (RM two-way ANOVA; sucrose main effect: *F*_(1,13)_ = 71.25, p < 0.0001; treatment main effect: *F*_(1,13)_ = 0.3502, *p* = 0.5641; sucrose × treatment interaction: F_(1,13)_ = 0.2342, *p* = 0.6365, followed by Sidak *post hoc* multiple comparison test). **(F)** Sucrose preference index in CE^P0-P10^ and PEE^P0-P10^ adult female mice during 1% sucrose preference test (unpaired *t*-test, *t*_(13)_ = 1.03, *p* = 0.3216). **(G)** Normalized consumption (g/Kg) of sucrose and water consumed during the 8% sucrose preference test in CE^P0-P10^ and PEE^P0-P10^ adult female mice (RM two-way ANOVA; sucrose main effect: *F*_(1,13)_ = 295.4, *p* < 0.0001; treatment main effect: *F*_(1,13)_ = 0.0943, *p* = 0.7637; sucrose × treatment interaction: *F*_(1,13)_ = 0.0659, *p* = 0.8014, followed by *post hoc* Sidak multiple comparison test). **(H)** Sucrose preference index in CE^P0-P10^ and PEE^P0-P10^ adult female mice during 8% sucrose preference test (unpaired *t*-test, *t*_(13)_ = 0.1221, *p* = 0.9047). **(I)** Normalized consumption (g/Kg) of sucrose and water consumed during the 1% sucrose preference test in CE^P0-P10^ and (Continued)FIGURE 3 (Continued)PEE^P0-P10^ adult male mice (RM two-way ANOVA; sucrose main effect: *F*_(1,21)_ = 53.91, *p* < 0.0001; treatment main effect: *F*_(1,21)_ < 0.0001, *p* = 0.9948; sucrose × treatment interaction: *F*_(1,21)_ = 0.1377, *p* = 0.7143, followed by Sidak *post hoc* multiple comparison test). **(J)** Sucrose preference index in CE^P0-P10^ and PEE^P0-P10^ adult male mice during 1% sucrose preference test (unpaired *t*-test, *t*_(21)_ = 0.4632, *p* = 0.648). **(K)** Normalized consumption (g/Kg) of sucrose and water consumed during the 8% sucrose preference test in CE^P0-P10^ and PEE^P0-P10^ adult male mice (RM two-way ANOVA; sucrose main effect: *F*_(1,22)_ = 373.9, *p* < 0.0001; treatment main effect: *F*_(1,22)_ = 0.3951, *p* = 0.5361; sucrose × treatment interaction: *F*_(1,22)_ = 0.3446, *p* = 0.5632, followed by *post hoc* Sidak multiple comparison test). **(L)** Sucrose preference index in CE^P0-P10^ and PEE^P0-P10^ adult male mice during 8% sucrose preference test (Mann–Withney *U* = 63, *p* = 0.6297). Data are expressed as mean ± SEM. Number of mice are indicated on each graph.

Animals used to model prenatal and PEE display deficits in affective behaviors, mainly related to anxiety-like (Bianco et al., 2021; Lopatynska-Mazurek et al., 2021b; Rouzer and Diaz, 2022) and depressive-like traits (Caldwell et al., 2008; Lopatynska-Mazurek et al., 2021b). To evaluate whether post-natal alcohol exposure would lead to similar deficits, we tested the PEE^P0-P10^ adult male and female progeny in the O-Maze test (Figure 3A), a high construct validity task to assess anxiety-like behavior in rodents (Walf and Frye, 2007; Braun et al., 2011). For these experiments, we used the adult offspring of cohorts #3, #4, and #5 at the age of P70-P260. First, we quantified the time spent in the open areas of the O-Maze and observed that, while males spent a longer time in the open areas compared to female mice, no difference was observed between CE^P0-P10^ and PEE^P0-P10^ progeny (Figure 3B). Second, we observed that while male subjects made more transitions in and out of open areas than females, CE^P0-P10^ and PEE^P0-P10^ adult offspring behaved similarly (Figure 3C). As in the three-chamber assay, no difference in distance moved was noted during the O-Maze task (Figure 3D). These data indicate that PEE^P0-P10^ does not induce significant anxiety-like deficits in either male or female adult offspring. To assess whether PEE^P0-P10^ impairs reward processing, we conducted a two-bottle sucrose preference test routinely used to evaluate anhedonia in rodents (Hoffman, 2016; Liu et al., 2018). Here, we tested the ability of the adult offspring to discriminate and consume a sucrose solution at two different concentrations during two consecutive 16-h periods. For these experiments, we used the adult offspring of cohorts #3, #4, and #5 in the age range P70-P260. In this test, CE^P0-P10^ and PEE^P0-P10^ female mice consumed more 1% sucrose solution than water (Figure 3E). There was a similar preference for the 1% sucrose solution in CE^P0-P10^ and PEE^P0-P10^ offspring (Figure 3F). We obtained similar results when the sucrose concentration was increased to 8%, with no differences in either sucrose consumption (Figure 3G) or preference (Figure 3H) between CE^P0-P10^ and PEE^P0-P10^ offspring. As in the female offspring, PEE^P0-P10^ male mice did not show deficits in sucrose 1% consumption (Figure 3I), sucrose 1% preference (Figure 3J), sucrose 8% consumption (Figure 3K), and sucrose 8% preference (Figure 3L). These data indicate that PEE does not induce significant abnormalities in hedonic/consummatory behavior, at least for a highly palatable carbohydrate in either male or female offspring.

### Postnatal ethanol exposure impairs the extinction of operant behavior in the adult female offspring

**Figure 4:**
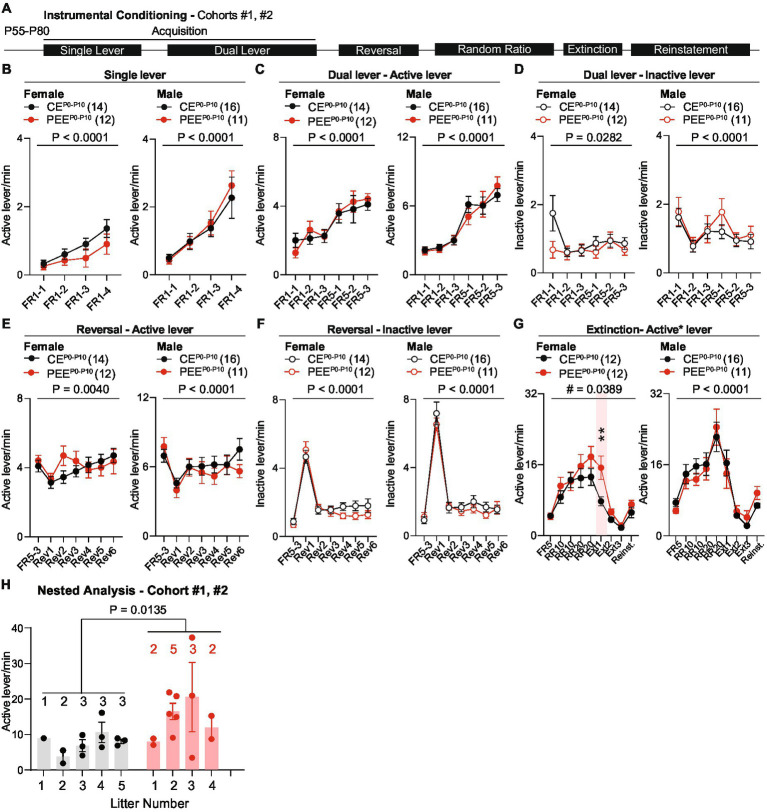
PEE impairs the extinction of operant behavior in the adult female offspring. **(A)** Timeline of behavioral experiments. **(B)** Active lever press frequency during single lever training in CE^P0-P10^ and PEE^P0-P10^ female and male mice (Female mice: RM two-way ANOVA; treatment main effect: *F*_(1,24)_ = 1.613, *p* = 0.2162; session main effect *F*_(3,72)_ = 12.84, *p* < 0.0001; treatment × session interaction: *F*_(3,72)_ = 0.87, *p* = 0.4608; Male mice: RM two-way ANOVA; treatment main effect: *F*_(1,25)_ = 0.0994, *p* = 0.7552; session main effect *F*_(3,75)_ = 16.74, *p* < 0.0001; treatment × session interaction: *F*_(3,75)_ = 0.2246, *p* = 0.879). **(C)** Active lever press frequency during dual lever training in CE^P0-P10^ and PEE^P0-P10^ female and male offspring (Female mice: RM two-way ANOVA; treatment main effect: *F*_(1,24)_ = 0.0435, *p* = 0.8365; session main effect *F*_(5,120)_ = 17.34, p < 0.0001; treatment × session interaction: *F*_(5,120)_ = 0.7638, *p* = 0.5777; Male mice: RM two-way ANOVA; treatment main effect: *F*_(1,25)_ = 0.0132, *p* = 0.9094; session main effect *F*_(5,125)_ = 38.69, p < 0.0001; treatment × session interaction: *F*_(5,125)_ = 0.7055, *p* = 0.6203). **(D)** Inactive lever press frequency during dual lever training in CE^P0-P10^ and PEE^P0-P10^ female and male mice (Female mice: RM two-way ANOVA; treatment main effect: *F*_(1,24)_ = 1.233, *p* = 0.2778; session main effect *F*_(5,120)_ = 2.608, *p* = 0.0282; treatment × session interaction: *F*_(5,120)_ = 2.234, *p* = 0.0552; Male mice: RM two-way ANOVA; treatment main effect: *F*_(1,25)_ = 0.4609, *p* = 0.5034; session main effect *F*_(5,125)_ = 6.081, p < 0.0001; treatment × session interaction: *F*_(5,125)_ = 0.5391, *p* = 0.7463). **(E)** Active lever press frequency during reversal of action-outcome associations in CE^P0-P10^ and PEE^P0-P10^ female and male offspring (Female mice: RM two-way ANOVA; treatment main effect: *F*_(1,24)_ = 0.1697, *p* = 0.684; session main effect *F*_(6,144)_ = 3.36, *p* = 0.004; treatment × session interaction: *F*_(6,144)_ = 1.914, *p* = 0.0823; Male mice: RM two-way ANOVA; treatment main effect: *F*_(1,25)_ = 0.3561, *p* = 0.556; session main effect *F*_(6,150)_ = 6.051, *p* < 0.0001; treatment × session interaction: *F*_(6,150)_ = 1.236, *p* = 0.2908). **(F)** Inactive lever press frequency during reversal training in CE^P0-P10^ and PEE^P0-P10^ female and male offspring (Female mice: RM two-way ANOVA; treatment main effect: *F*_(1,24)_ = 0.6529, *p* = 0.427; session main effect *F*_(6,144)_ = 64.74, *p* < 0.0001; treatment × session interaction: *F*_(6,144)_ = 1.305, *p* = 0.2584; Male mice: RM two-way ANOVA; treatment main effect: *F*_(1,25)_ = 0.3105, *p* = 0.5823; session main effect *F*_(6,150)_ = 119.7, *p* < 0.0001; treatment × session interaction: *F*_(6,150)_ = 0.7679, *p* = 0.5963). **(G)** Active lever press frequency during random ratio training, extinction, and reinstatement of action-outcome associations in CE^P0-P10^ and PEE^P0-P10^ female and male offspring (Female mice: RM two-way ANOVA; treatment main effect: *F*_(1,22)_ = 2.255, *p* = 0.1474; session main effect *F*_(8,176)_ = 31.71, *p* < 0.0001; treatment × session interaction: F_(8,176)_ = 2.092, *p* = 0.0389; between-group Sidak *post hoc* test, ** = 0.0077; Male mice: RM two-way ANOVA; treatment main effect: (Continued)FIGURE 4 (Continued)*F*_(1,25)_ = 0.0137, *p* = 0.9079; session main effect *F*_(8,200)_ = 24.76, *p* < 0.0001; treatment × session interaction: *F*_(8,200)_ = 0.7263, *p* = 0.6682). **(H)** Nested analysis of active lever press frequency in CE^P0-P10^ and PEE^P0-P10^ female offspring on the first day of extinction (nested *t*-test, *t*_(22)_ = 2.685, *p* = 0.0135). Data are expressed as mean ± SEM. Number of mice are indicated on each graph.

We then assessed the impact of PEE^P0-P10^ on instrumental conditioning during adulthood. These experiments included the offspring of cohorts #1 and #2 and began when animals reached an age of P55-P80. We trained animals in a 4-day single-lever schedule to obtain one sucrose reward (FR1), followed by dual-lever training when an inactive lever was introduced and counterbalanced across mice. This phase consisted of 3 days of FR1 training followed by 3 days on an FR5 schedule. At the end of the acquisition period, animals underwent an FR5 reversal learning phase when active and inactive levers were switched. After six training sessions, animals were retrained on a random-ratio (RR) reinforcement schedule followed by extinction when lever pressing was no longer reinforced (Figure 4A). Neither PEE^P0-P10^ female nor male mice show deficits in active lever press frequency during single lever training (Figure 4B) or in active (Figure 4C) and inactive lever press frequency (Figure 4D) during dual-lever training. Across reversal learning sessions, both CE^P0-P10^ and PEE^P0-P10^ male and female offspring increased their active lever press frequency to the same levels as the last acquisition day (Figure 4E) and decreased inactive lever press frequency (Figure 4F). During random ratio training, female PEE^P0-P10^ mice showed no difference in active lever press frequency but heightened active lever press frequency during extinction day 1 (Figure 4G), with no statistically significant difference in reinstatement (Figure 4G). Active lever press frequency during random ratio, extinction, and reinstatement was similar between CE^P0-P10^ and PEE^P0-P10^ adult male offspring (Figure 4G). To exclude that extinction deficits derived from multiple representations of litter exposure due to including more than one pup per litter, we performed a nested analysis that considers each pup as a biological replicate of each litter. This per-litter analysis confirms that the PEE^P0-P10^ female offspring have a higher lever press frequency on extinction day 1 than the CE^P0-P10^ offspring (Figure 4H). These data reveal a sex-specific persistence of action-outcome associations in PEE^P0-P10^ adult female, but not male, offspring.

## Discussion

This study aimed to investigate the socioemotional and cognitive deficits in the progeny of a PEE^P0-P10^ mouse model. PEE^P0-P10^ adult female, but not male, offspring show altered social approach and impaired extinction of instrumental action-outcome associations. These sex-specific socioemotional and cognitive deficits were not due to significant abnormalities in locomotor activity, exploration of the open areas in the O-maze task, or sucrose preference in a two-bottle choice paradigm, which indicate the absence of major anxiety-like or anhedonic states. However, some considerations are warranted.

In the present study, we included five cohorts of mice postnatally exposed to alcohol, which reached an average BAC of 150 mg/dL. This concentration is well above the intoxication threshold of 80 mg/dL and induces severe behavioral symptoms in humans (Koob et al., 2014). The combination of cyclic exposures (P0-P3, P6-P10) and the high BAC relate to the binge-like patterns of gestational alcohol use observed in about 3% of the general pregnant population in European, African and American countries (Popova et al., 2018). This exposure pattern induced a sex-specific reduction in the body weight of female PEE^P0-P10^ offspring, resembling anatomical dysmorphologies seen in humans exposed to high alcohol levels during development (Mattson and Riley, 1998). Whether changes in corticosterone, growth hormone, and testosterone (Gabriel et al., 1998) levels underlie these defects in the female PEE^P0-P10^ offspring remains an open question.

Individuals with FASD (Kully-Martens et al., 2012) and the male progeny of animal models of PEE show dyadic social interaction and recognition deficits during adulthood (Boschen et al., 2014; Subbanna et al., 2018; Joshi et al., 2019; Shivakumar et al., 2021). We observed that PEE^P0-P10^ male and female offspring have intact sociability, expressed as longer time spent in the unfamiliar social vs. object chamber (Yang et al., 2011) and similar locomotor activity during the three-chamber sociability test. However, our automated analysis of exploratory behavior revealed a clear preference for the distal vs. proximal location relative to the same-sex conspecific in PEE^P0-P10^ females, which was not observed in CE^P0-P10^ adult females. These impairments might be interpreted as a mild social avoidance phenotype. Previous studies defined strain-specific social avoidance in female mice as a relative reduction in exploratory behavior in the presence vs. absence of a social stimulus (Brodkin et al., 2004). In mice, social avoidance can also be quantified as the relative reduction in the time spent in proximal vs. distal areas to a social stimulus after a chronic social defeat paradigm (Harris et al., 2018). Thus, one hypothesis might be that the unfamiliar conspecific might represent an aversive stimulus for the adult PEE^P0-P10^ female progeny, which would acquire an even stronger negative valence upon chronic social stress paradigms.

During the three-chamber sociability test, we also observed a longer latency to first approach the novel social stimulus in the PEE^P0-P10^ compared to the CE^P0-P10^ progeny, which might indicate novelty-induced avoidance. Notably, previous models of prenatal (Rouzer et al., 2017) and adolescent alcohol exposure followed by acute restrain stress (Kasten et al., 2020) reported novelty-induced hypophagia in male and female subjects, respectively. These data highlight the possibility that the adult offspring of PEE^P0-P10^ might also display negative affective states exacerbated by novel stimuli of different nature.

Depending on the exposure paradigm, PEE causes anxiety-like behavior in male and female rats (Bianco et al., 2021; Lopatynska-Mazurek et al., 2021b), reminiscent of co-morbid anxiety symptoms observed in FASD patients (Famy et al., 1998; Barr, 2006; Pei et al., 2011). We did not observe significant anxiety-like behavior during the elevated O-maze test in the adult PEE^P0-P10^ mouse offspring. However, it is crucial to recognize that previous authors reported anxiety-like behavior in prenatal and PEE progeny (Rouzer et al., 2017; Bianco et al., 2021; Lopatynska-Mazurek et al., 2021b; Rouzer and Diaz, 2022) using different behavioral paradigms, including the open field test (Bianco et al., 2021), elevated plus maze (Dursun et al., 2006; Cullen et al., 2013), light–dark box test (Cullen et al., 2013), and novelty-induced hypophagia (Rouzer et al., 2017; Bianco et al., 2021). Thus, additional behavioral experiments might reveal anxiety-like traits in the adult PEE^P0-P10^ male and female offspring. As discussed earlier, the increased time spent in distal areas to a novel social stimulus and the increased latency to social approach might indicate anxiety-like traits related to social interaction. Whether social avoidance phenotypes are due to the novel and anxiogenic nature of the stimulus or can be exacerbated by post-natal stressors (Hellemans et al., 2008) will be addressed in future studies.

Similar to anxiety, depression is a comorbidity often observed in individuals who received an FASD diagnosis (Pei et al., 2011). One core component of depression is reduced hedonic behavior (Fava and Kendler, 2000; Pizzagalli et al., 2008), which can be evaluated using a two-bottle choice test in rodents (Liu et al., 2018). In this study, we specifically assessed whether the adult PEE^P0-P10^ male and female progeny would prefer a sucrose solution (at two different concentrations) over water and failed to find any deficits. This allows us to draw two important conclusions: on the one hand, PEE^P0-P10^ does not alter reward-processing mechanisms, and on the other, it implies that the adult male and female progeny do not show anhedonia-like behavior indicative of a depressive-like phenotype in this test. However, it is crucial to consider that in rodents, core components of depressive-like states can be assessed and revealed by using other behavioral tests, including the forced-swim task (Porsolt et al., 1978; Lopatynska-Mazurek et al., 2021b), the tail-suspension test (Cryan et al., 2005), the learned helplessness test (Seligman, 1972; Pryce et al., 2012), and the urine scent marking test (Lehmann et al., 2013). Whether depressive like-states might emerge in the male and female PEE^P0-P10^ progeny during these tests remains a future investigation venue.

Beyond socioemotional impairments, FASD individuals and animals used as models of PEE display deficits in several aspects of executive function (Bariselli and Lovinger, 2021). As opposed to previous Y-Maze, Barnes Maze, and Morris Water Maze tasks conducted on the progeny of PEE rats and mice (O’Leary-Moore et al., 2006; Allan et al., 2014; Subbanna et al., 2018; Joshi et al., 2019; Gibula-Tarlowska et al., 2021; Shivakumar et al., 2021; Lopatynska-Mazurek et al., 2021a; Risbud et al., 2022), we failed to observe deficits in associative and reversal learning during operant training. Several factors may account for these discrepancies. For example, the developmental timing of alcohol exposure could be an important variable. While previous studies used a prenatal alcohol exposure paradigm (Allan et al., 2014; Marquardt et al., 2014), we utilized a post-natal alcohol exposure protocol. The type of behavioral assay may also underlie the difference in results. While previous authors assessed spatial navigation-based associative learning (O’Leary-Moore et al., 2006; Allan et al., 2014; Subbanna et al., 2018; Joshi et al., 2019; Gibula-Tarlowska et al., 2021; Shivakumar et al., 2021; Lopatynska-Mazurek et al., 2021a; Risbud et al., 2022) or cue-based reversal learning tasks (Marquardt et al., 2014), we examined self-paced instrumental reversal learning where non-cued active and inactive levers were switched. Finally, the age of the experimental subjects at testing is an important variable. Previous experiments showed reversal learning deficits in juvenile, but not adult, rats postnatally exposed to alcohol (O’Leary-Moore et al., 2006), while we used adult subjects only.

After reversal learning, adult female subjects were re-trained using a random ratio (RR) schedule that, in previous experiments, resulted in hyper-responding in mice exposed to ethanol throughout the prenatal and early postnatal periods (Cuzon Carlson et al., 2020). In this study, we limited our PEE to postnatal periods that might not be enough to increase lever-pressing behavior. However, we noticed that reward omission during the extinction phase resulted in heightened lever-pressing behavior in the PEE^P0-P10^ compared to the CE^P0-P10^ adult female offspring, demonstrating the persistence of action-outcome responding. These defects highlight behavioral maladaptations to changes in environmental contingencies (Bouton, 2004) following PEE. Once again, the absence of extinction defects associated with reinstatement deficits reported in prenatally alcohol-exposed subjects (Olguin et al., 2019) might highlight fundamental differences in the teratogenic effects of alcohol on the developing brain.

Our experiments reveal specific behavioral deficits in the adult female progeny of a postnatal mouse model of PEE^P0-P10^, mainly related to the social and behavioral adaptation domains. One limitation of this study is the need for more data on the phase of the estrous cycle of the female offspring, which influence both social (Ervin et al., 2015) and operant (Verharen et al., 2019) behavior in rodents. Together with the existing literature on different models of fetal alcohol exposure, these data highlight the importance of timing of exposure in mediating the teratogenic effects of alcohol on the developing brain. This approach will help to identify and tailor specific therapeutic interventions for subgroups of individuals affected by FASD.

## Data availability statement

The raw data supporting the conclusions of this article will be made available by the authors, without undue reservation.

## Ethics statement

The animal study was reviewed and approved by Animal Care and Use Committee of the NIAAA Division of Intramural Clinical and Biological Research.

## Author contributions

SB, YM, and DL conceived the study. SB wrote the manuscript with the assistance of the other authors. NR performed instrumental learning experiments and helped with the generation of PEE^P0-P10^ mice. NR and NW performed the sociability, elevated O-maze, and sucrose preference test. All authors contributed to the article and approved the submitted version.

## Funding

This research was supported by the Intramural Research Program of the NIH (ZIAAA000416). SB is supported by the Center on Compulsive Behaviors at NIH.

## Conflict of interest

The authors declare that the research was conducted in the absence of any commercial or financial relationships that could be construed as a potential conflict of interest.

## Publisher’s note

All claims expressed in this article are solely those of the authors and do not necessarily represent those of their affiliated organizations, or those of the publisher, the editors and the reviewers. Any product that may be evaluated in this article, or claim that may be made by its manufacturer, is not guaranteed or endorsed by the publisher.
